# Factors affecting prolonged time to extubation in patients given remimazolam

**DOI:** 10.1371/journal.pone.0268568

**Published:** 2022-05-18

**Authors:** Yoko Shimamoto, Michiyoshi Sanuki, Shigeaki Kurita, Masaya Ueki, Yoshie Kuwahara, Ayumu Matsumoto

**Affiliations:** Department of Anesthesiology, NHO Kure Medical Center, Kure, Hiroshima, Japan; Cleveland Clinic, UNITED STATES

## Abstract

**Purpose:**

To analyze the cause of prolonged recovery from general anesthesia with remimazolam.

**Methods:**

We studied 65 patients under general anesthesia with remimazolam. According to time to extubation, patients were divided into short period (SP) (n = 34, < 15 min) and long period (LP) (n = 31, ≥ 15 min) groups. Variables affecting time to extubation such as age, sex, height, body weight, body mass index (BMI), plasma albumin concentration, ASA class, duration of surgery, and total duration of general anesthesia, and total dose of remimazolam were compared between SP and LP groups. At the end of remimazolam infusion and upon extubation, predictive remimazolam concentrations were calculated using pharmacokinetic/pharmacodynamic three compartment modeling.

**Results:**

LP group showed significantly higher BMI, older age, and lower plasma albumin concentration compared with those of SP group. Logistic regression analysis showed that the probability of time to extubation of ≥ 15 min was higher in patients with BMI greater than 22.0 kg/m^2^ (AUC 0.668, 95% CI 0.533‒0.803), ages older than 79.0 years (AUC 0.662, 95% CI 0.526‒0.798), and plasma albumin concentrations lower than 3.60 g/dl (AUC 0.720, 95% CI 0.593‒0.847). LP group showed significantly lower predicted remimazolam concentration than SP group upon extubation despite no difference in concentration between both groups at the end of infusion. Pharmacological analysis estimates that LP group is more sensitive to remimazolam than SP group through amplified responses.

**Conclusions:**

Lower remimazolam doses should be considered for the overweight patients, elderly, and those with lower plasma albumin concentration.

## Introduction

Remimazolam besilate is an ultra-short acting benzodiazepine being used for the induction and maintenance of general anesthesia. In January 2020, remimazolam was approved as a general anesthetic agent in Japan for the first time in the world. Remimazolam is also undergoing regulatory assessment for this indication in South Korea and for use in procedural sedation in the USA, EU, and China. In 2007, Kilpatrick et al. [1] reported that remimazolam (CNS7056) has sedative effects on rodents, inducing a reduction in locomotor activity at lower doses and a loss-of-righting reflex. In 2012, Antonik et al. [2] published a study on 81 healthy volunteers that evaluated the safety, pharmacokinetics, and pharmacodynamics of remimazolam (CNS7056). Remimazolam is a full agonist of the benzodiazepine binding site of the gamma-aminobutyric acid (GABA)_A_ receptor. It is rapidly metabolized by esterases, and its simulated context-sensitive half-time after infusion for 4 h was 6.8 ± 2.4 min [3]. Yamamoto et al. [4] have reported a case of re-sedation after reversal of remimazolam using flumazenil, an antagonist of benzodiazepine sedatives. Further clinical studies are therefore needed for assessing the pharmacological properties of remimazolam.

One performance metric used by anesthesiologists is the incidence of prolonged time to endotracheal extubation, which refers to extubations that occur 15 min or later after the end of surgery on post-operative patient [5, 6]. Several patient-related, anesthesia-related, and procedure-related factors are independently associated with a prolonged time to extubation.

The present study aimed to determine various factors that may contribute to prolonged time to extubation in patients administered remimazolam. This information can help in formulating dosage recommendations of remimazolam in special surgical populations such as the elderly and overweight patients.

## Materials and methods

### Patients

The study population included 65 Japanese patients (32males, mean age 69.2 ± 17.3 years, range 16 to 96 years) who underwent elective surgery under general anesthesia at NHO Kure Medical Center, Japan between August 2020 and November 2020. Written informed consent was obtained from all patients. Demographic, clinical, and infusion data were entered and analyzed in the department of anesthesiology information system. The Institutionary Review Board of the NHO Kure Medical Center approved the retrospective analysis of clinically acquired data and this study was approved by Ethics Committees (2020‒59, UMIN000046549).

Prolonged time to extubation has been defined as 15‒min or longer interval from the end of surgery to the removal of the tracheal tube [5, 6]. Our patients were divided into two groups according to the time to extubation (defined as the time from the end of remimazolam infusion to extubation); short period (SP) group (n = 34) and long period (LP) group (n = 31). The SP group was defined as those having a time to extubation of < 15 min, and the LP group, as having that of ≥ 15 min. The following prespecified variables were evaluated: age, sex, height, body weight, body mass index (BMI), plasma albumin concentration, American Society of Anesthesiologists (ASA) class, duration of surgery, total duration of general anesthesia, and total dose of remimazolam. Since the time to extubation was affected by the administration of flumazenil, the LP group was divided into two subgroups: the LPF(-) group (n = 15) without flumazenil and the LPF(+) group (n = 16) with flumazenil.

### General anesthesia

General anesthesia was induced by intravenous bolus of remimazolam doses at 1 mg/kg, remifentanil at 0.3 to 0.5 μg/kg/min, and rocuronium at 0.6 to 0.9 mg/kg. Following loss of consciousness, patients subsequently received remimazolam at infusion rates of 0.2 to 1.0 mg/kg/h as maintenance dose, and adjusted to maintain adequate sedation (patients state index, PSI, 30 to 50) with or without regional anesthesia. Associated with emergence from remifentanil analgesia, fentanyl was perioperatively administered as alternative analgesic therapy for postoperative pain control.

After surgical procedure completions, remimazolam was discontinued and 2 ‒ 4 mg/kg of sugammadex was used to reverse neuromuscular blockade. Complete return of neuromuscular function from general anesthesia was ensured using quantitative monitoring of the degree of the residual blockade, with an adductor pollicis train-of-four ratio at least 0.90. Since co-administration of opioids resulted in synergistic potentiation of remimazolam-induced sedation and respiratory depression, the decision regarding whether flumazenil was to be administered was made based on the following conditions: 1) spontaneous breathing ≥ 8 breaths/min, defined as the absence of opioid-induced respiratory depression in the unconscious state, 2) spontaneous breathing < 8 breaths/min, in principle, with predicted effect-site concentration of remifentanil or fentanyl ≤ 2 ng/mL, and ≤ 1.5 ng/mL in the elderly. Remimazolam was reversed with flumazenil in 16 LP patients and none of the SP patients. Awake extubation was performed following confirmation of eye opening, obeying commands, adequate spontaneous ventilation, hemodynamic stability, normothermia, adequate analgia, and reversal of neuromuscular blockade.

### Analysis of pharmacokinetic (PK)/pharmacodynamic (PD) modeling for remimazolam

At the end of remimazolam infusion and upon extubation, predictive plasma concentration of remimazolam was calculated using PK/PD three compartment modeling for remimazolam based on Runge–Kutta method (Excel PkPd Ver1.43, http://home.hiroshima-u.ac.jp/r-nacamura/) (Nakamura R.) [7]. Parameters of the PK/PD model were sex, weight, height, and age.

### Statistics

Data are given as median (IQR, 25%—75% interquartile range) and mean ± standard deviation. Statistical analysis was performed using EZR (Saitama Medical Center, Jichi Medical University, Saitama, Japan), a graphical user interface for R (The R Foundation for Statistical Computing, Vienna, Austria). It is a modified version of R commander designed to add statistical functions frequently used in biostatistics [8].

Mann-Whitney U test, Kruskal-Wallis test, and Bonferroni test were used to compare the variables as appropriate. Categorical variables were compared using Fisher’s exact test. Multiple logistic regression analysis was performed to determine possible predictors of prolonged time to extubation. A receiver–operating characteristic (ROC) curve was plotted for each predictor, and the area under the curve was calculated as an index for the time to extubation. P < 0.05 was considered to be statistically significant.

## Results

### Analysis between the SP and LP groups

Patients’ demographics are shown in Table 1.

**Table 1:** Clinical characteristics of patients in LP and SP groups.

Variables	SP	LP	P value
Patients, n	34	31	
Male sex, n (%)	18 (52.9)	14 (45.2)	0.622
Age, years	69.5 (57–78)	80 (63–85)	**0.025** *
Height, cm	158.8 (151.7–166.4)	157 (147–162)	0.348
Body weight, kg	57.3 (48.4–65.6)	60.7 (50.1–75.5)	0.287
BMI, kg/m^2^	22.4 (20.2–25.3)	25.5 (22.2–29.5)	**0.019** *
Plasma albumin, g/dl	4.0 (3.2–4.3)	3.2 (2.7–3.6)	**0.002** *
ASA class (IQR)	2 (2 ‒ 2)	2 (2 ‒ 3)	0.061
Duration of surgery, min	146 (93–204)	118 (47–233)	0.393
Total duration of general anesthesia, min	193 (135–256)	169 (114–331)	0.932
Total dose of remimazolam administered, mg	75.4 (47.8–110.0)	67.1 (44.1–119.0)	0.958
Administration of flumazenil, n (%)	0	16 (51.6)	

Data are median (IQR; 25% ‒ 75% interquartile range).

*significant difference.

SP, short period (time to extubation < 15 min); LP, long period (time to extubation ≥ 15 min); BMI, body mass index; ASA, American Society of Anesthesiologists.

**Fig 1:**
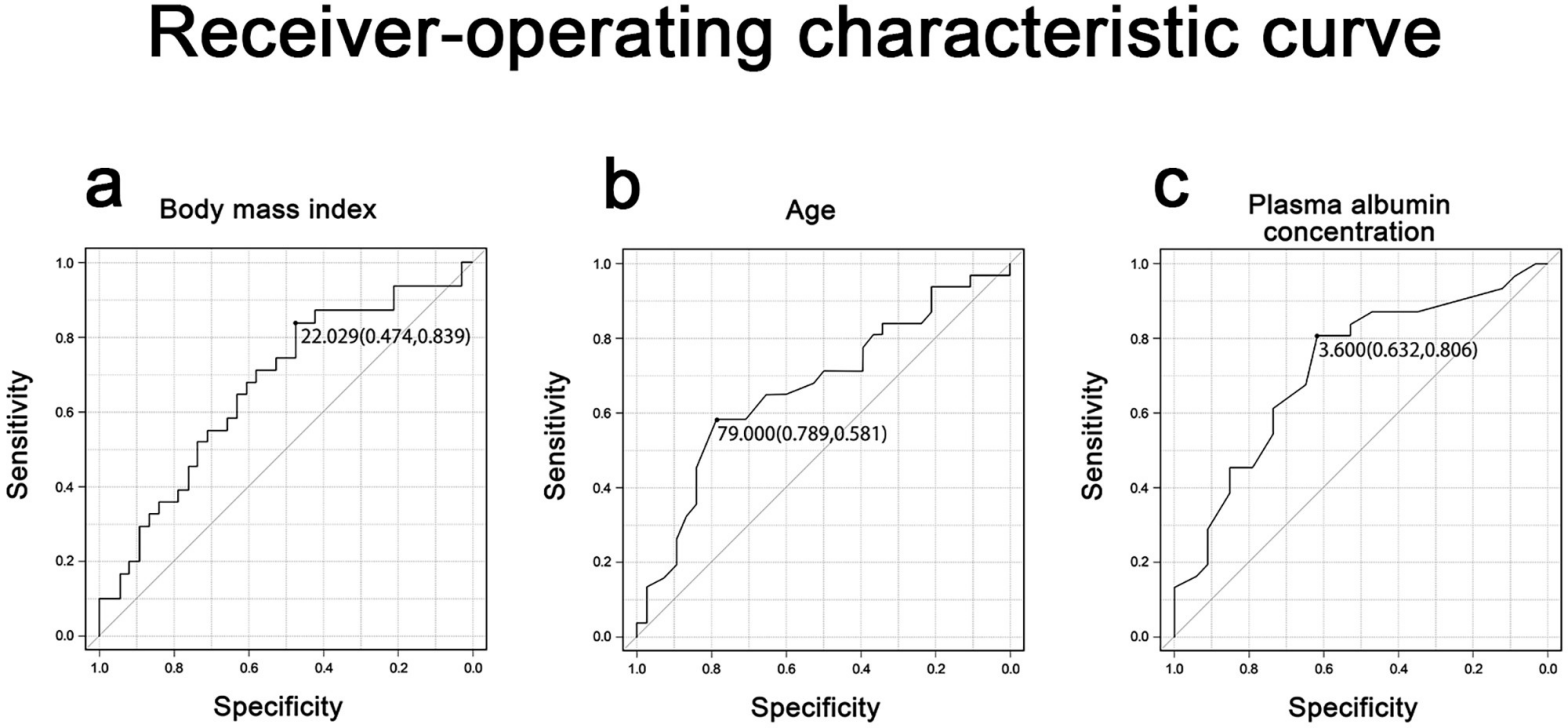
Receiver‒operating characteristic curve. (a) Time to extubation ≥ 15 min for body mass index (BMI) over 22.0 kg/m^2^. (b) Time to extubation ≥ 15 min for age over 79.0 years. (c) Time to extubation ≥ 15 min for plasma albumin concentration below 3.60 g/dl.

The LP group showed a significantly higher BMI, older age, and lower plasma albumin concentration compared with those observed in the SP group. Logistic regression analysis showed that probability of a time to extubation of ≥ 15 min was higher in patients with BMI greater than 22.0 kg/m^2^ (AUC 0.668, 95% CI 0.533‒0.803) (Fig 1a), age older than 79.0 years (AUC 0.662, 95% CI 0.526‒0.798) (Fig 1b), and plasma albumin concentration lower than 3.60 g/dl (AUC 0.720, 95% CI 0.593‒0.847) (Fig 1c). Sex, height, body weight, ASA class, duration of surgery, total duration of general anesthesia or the total dose of remimazolam administered was found to have no effect.

There was no significant difference in predicted remimazolam concentration between the SP and LP groups at the end of infusion. Conversely, the LP group had a significantly lower predicted remimazolam concentration than the SP group upon extubation (Table 2).

**Table 2:** Predicted plasma concentration of remimazolam in LP and SP groups.

Variables	SP	LP	P value
Patients, n	31	34	
At the end of remimazolam infusion, μg/ml	0.278 (0.218–0.368)	0.277 (0.198–0.355)	0.371
At the time of extubation, μg/ml	0.146 (0.107–0.214)	0.101(0.070–0.139)	**0.000** *

Data are median (IQR; 25% ‒ 75% interquartile range).

*significant difference.

SP, short period (time to extubation < 15 min); LP, long period (time to extubation ≥ 15 min).

Although there is a presumption that a longer time to extubation induces greater reduction in predicted remimazolam concentration, another important finding is that the LP group took more time to reach lower predicted remimazolam concentration for awake extubation, compared to that required by the SP group. These results suggest increased sensitivity to remimazolam in the LP group than in the SP group.

**Fig 2:**
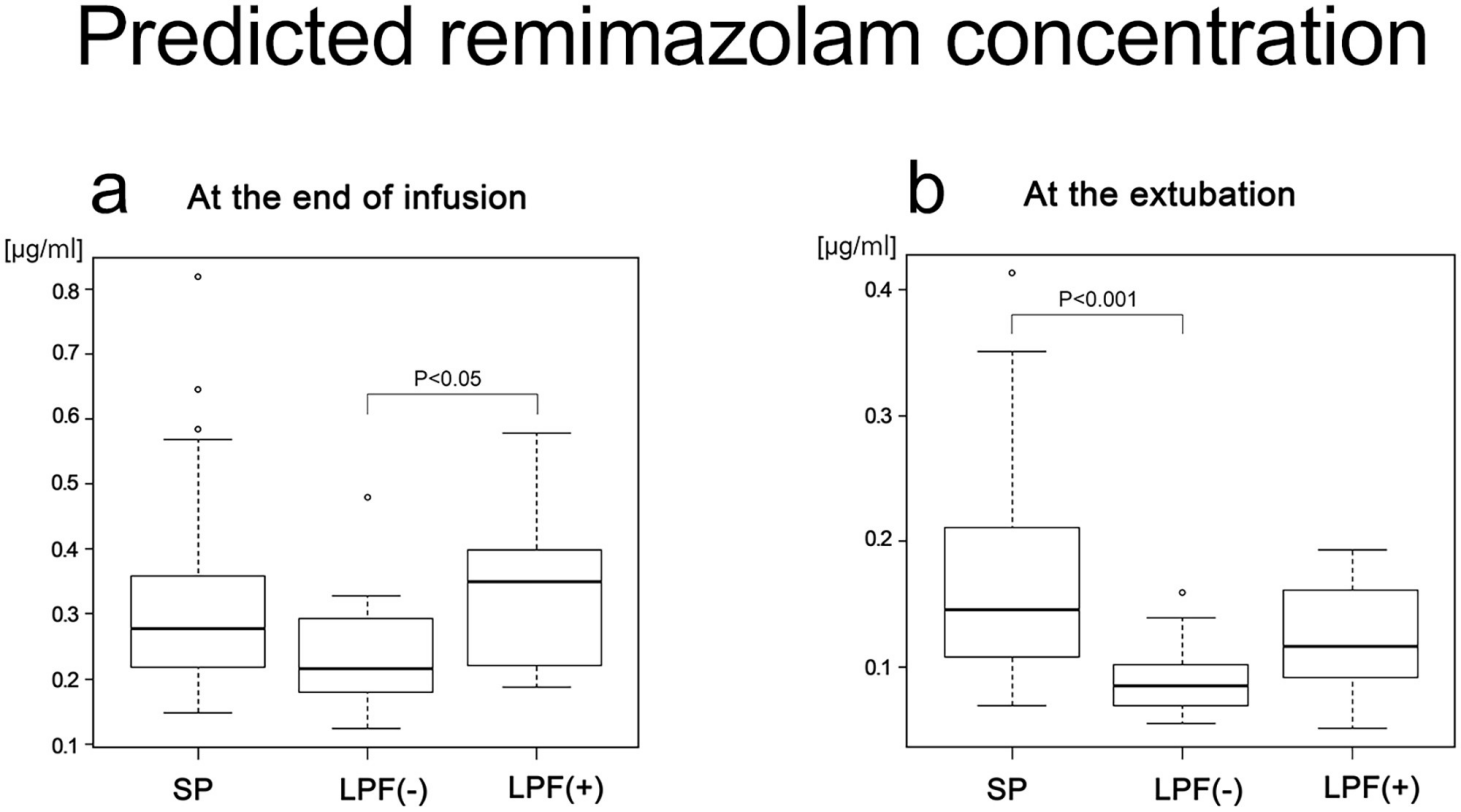
Predicted remimazolam concentration. (a) at the end of infusion. (b) at the extubation. SP, short period group; LPF(-), long period group without flumazenil; LPF(+), long period group with flumazenil.

Flumazenil was administered to 16 patients in the LP group and none on the SP group. For patients who received flumazenil, the time to extubation with remimazolam was censored indicated above. Thus, the LP group was subdivided into two subgroups: LPF(-) without flumazenil (n = 15) and LPF(+) with flumazenil (n = 16). None of the LPF(+) group required repeat doses of flumazenil. Predicted remimazolam concentration was compared among the SP, LPF(-), and LPF(+) groups. At the end of infusion, predicted remimazolam concentration was higher in the LPF(+) group than in the LPF(-) group (Fig 2a), but there was no difference in the predicted remimazolam concentration between the SP and LPF(-) groups. At the time of extubation, only the LPF(-) group demonstrated lower predicted remimazolam concentration than that of the SP group, but the LPF(+) group did not (Fig 2b).

### Pharmacological analysis of predicted remimazolam concentration during the period from the end of infusion to extubation

Concerning PK/PD analysis, we converted predicted remimazolam concentration to a log scale and compared the SP and LPF(-) groups (Table 3).

**Table 3:** Log predicted remimazolam concentration in SP and LPF(-) groups.

Variables	SP	LPF(-)	P value
Patients, n	34	15	
At the end of remimazolam infusion, log M	-6.33 (-6.44—-6.21)	-6.44 (-6.53—-6.30)	0.112
At the end of extubation, log M	-6.61 (-6.74—-6.45)	-6.85 (-6.95—-6.76)	**0.000** *
Difference of concentration, log M	0.26 (0.21–0.33)	0.41 (0.33–0.47)	**0.000** *

Data are median (IQR; 25% ‒ 75% interquartile range).

*significant difference.

SP, short period (time to extubation < 15 min); LPF(-), long period (time to extubation ≥ 15 min) without flumazenil.

**Fig 3:**
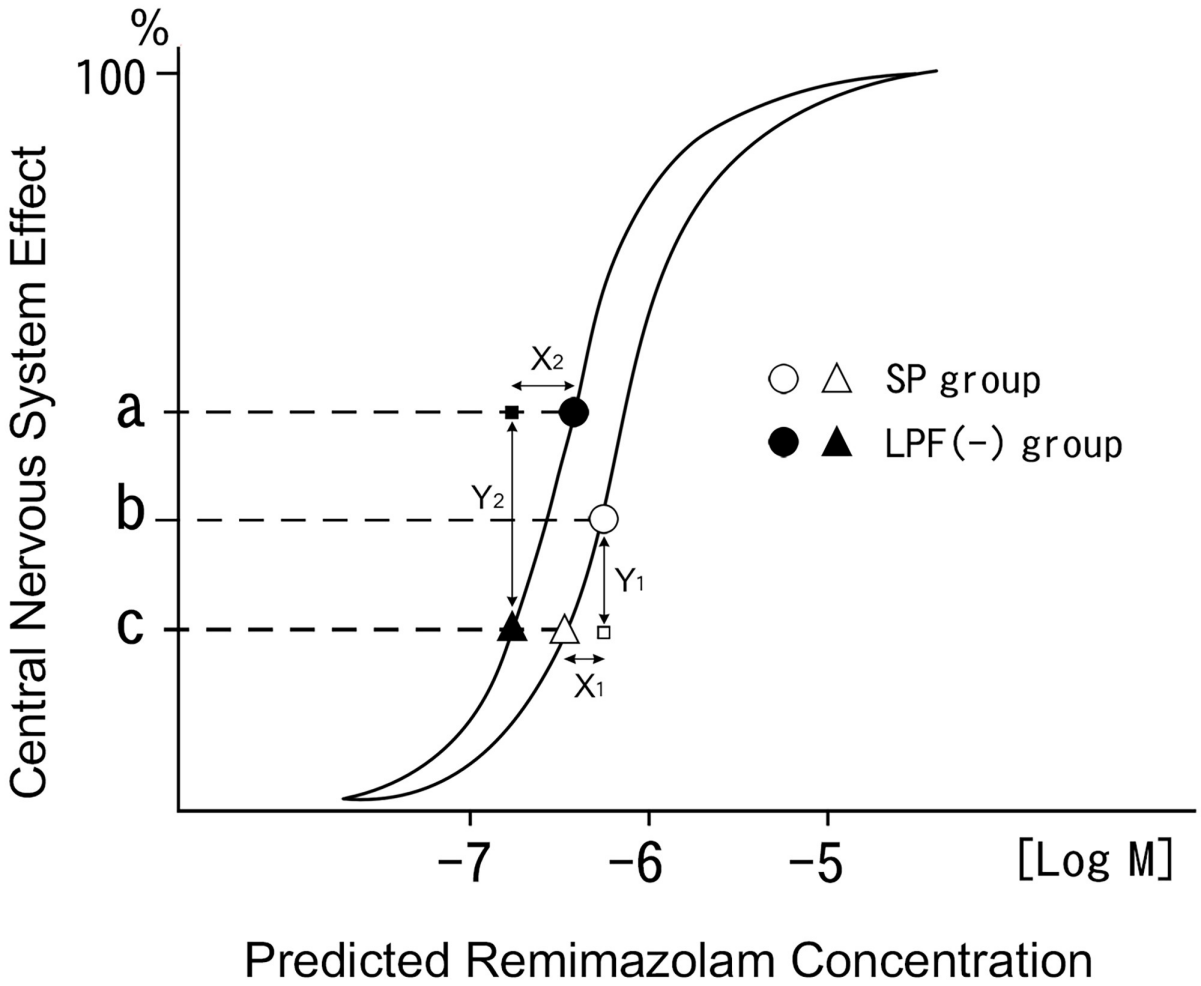
Schematic representation of the dose-response curve; log predicted remimazolam concentration on X-axis, and linear central nervous system effect on Y-axis. The dose-response curve in the SP group was shifted to the left in the LPF(-) group. a: Central nervous system effect at the end of infusion in the LPF(-) group. b: Central nervous system effect at the end of infusion in the SP group. c: Central nervous system effect at the extubation in the SP and LPF(-) groups. Open circle and open triangle: SP group. Filled circle and filled triangle: LPF(-) group. X_1_ and X_2_ refer to the difference between the log predicted concentration at the end of infusion and that at the extubation in the SP and LPF(-) groups, respectively. X_2_ is significantly greater than X_1_. Y_1_ and Y_2_ refer to the change in response between the SP and LPF(-) groups, respectively. Since the triangleΔ○□ and the triangle ●▲■ are similar figures. The ratio of Y_2_ to Y_1_ is determined by a following formula, Y_2_/Y_1_ = X_2_ /X_1_. Because X_2_ is significantly greater than X_1_, Y_2_ is estimated to be larger than Y_1_. SP, short period group; LPF(-), long period group without flumazenil.

When the SP and LPF(-) groups became awake enough to be extubated and have regained the same central nervous system effect, the LPF(-) group demonstrated significantly lower log predicted concentration of remimazolam than the SP group. This indicates that the LPF(-) group is more sensitive to remimazolam than the SP group. The LPF(-) group had an amplified response to remimazolam compared with that of the SP group, as evident from the shifting of the dose-response curve to the left (Fig 3).

However, there was no difference in the log predicted remimazolam concentration between the SP and LPF(-) groups at the end of infusion. Therefore, the LPF(-) group had a significantly greater difference between the log predicted remimazolam concentration at the end of infusion and that upon extubation compared to that of the SP group (p = 2.55e-05) (Table 3). Because this difference in log predicted concentration is supposed to be proportional to that in the central nervous system effect, the LPF(-) group was considered to produce a larger change in response to remimazolam than the SP group, suggesting a comparatively excess dosing of remimazolam in the LPF(-) group (Fig 3).

## Discussion

The selection of anesthetic technique and anesthetic drugs determines the duration of unconsciousness. In intravenous general anesthesia, the duration of unconsciousness is influenced by context-sensitive half-time of drug, total dose of drug, and co-administration with other drugs. One objective of this analysis was to evaluate whether increased duration of surgery or total remimazolam dosing delayed the recovery rate after general anesthesia. Notably, the time to extubation was not affected by the duration of surgery, total duration of anesthesia or total dose of remimazolam, which may be explained by the short context-sensitive half time of remimazolam.

### Factors affecting the time to extubation

The current analysis revealed that higher BMI, older age and lower plasma albumin concentration resulted in a longer time to extubation.

#### Increased BMI

Higher BMI increases the time to extubation independently of age and plasma albumin concentration (p = 0.827, p = 0.611, respectively) in this study. Zhou et al. established the population PK-PD model of remimazolam for evaluating dosing regimens in general anesthesia and showed the adequacy of weight-dependent dosing for this drug [9]. Whereas, Antonik et al. [2] demonstrated that in healthy volunteers (weight: 60–100 kg, BMI: 18–30 kg/m^2^), there was no clear association between weight and systemic remimazolam clearance; a 1 min intravenous infusion of remimazolam (0.01–0.35 mg/kg) was administered once to patients in their clinical trial. We investigated long-duration general anesthesia by the administration of an intravenous bolus and subsequent infusion of remimazolam as a maintenance dose. Thus, the discrepancy in results may be due to differences in the method of administering remimazolam in different studies and countries.

The difference between the total body weight and lean body mass increases as the total body weight increases [10]. Thus, overweight patients have an increased amount of adipose tissue as compared to that of non-overweight patients. The volume of distribution of a drug demonstrates an estimate of the extent to which a drug is distributed in extravascular tissues. Remimazolam is a lipophilic benzodiazepine with a high volume of distribution into excess adipose tissue, resulting in a prolonged half-life in the overweight patients. Although larger initial doses of this drug may be required in the overweight patients because of the increased volume of distribution, the potential for supratherapeutic concentrations following appreciable accumulation of this drug may occur despite weight-dependent dosing even after ending the remimazolam administration. In this study, logistic regression analysis showed that a time to extubation ≥ 15 min was higher in patients with BMI > 22.0 kg/m^2^. This suggests that dose adjustments might be needed in overweight patients. This produces concern that excessively deep anesthesia may result after the end of remimazolam administration for overweight patients if the dosage is based only on the total body weight.

Furthermore, overweight affects almost every other system in the body such as cardiac function, regional tissue blood flow, plasma protein binding, and drug elimination process which may also impact drug distribution. Regarding plasma protein binding, overweight does not seem to have an influence on drug binding to albumin. This is shown by the lack of a relationship between BMI and plasma albumin concentration in this study. Remimazolam is largely and quickly metabolized in the liver that very little of the parent drug is recovered from plasma or urine following intravenous administration. It has been observed in overweight patients that fatty liver and its relatively low tissue perfusion, accompanied by presence of altered cardiac performance, may possibly reduce drug elimination. These changes may influence the recommended dosages of remimazolam used in general anesthesia.

Manufacturer dosing is recommended on a per–kg basis according to the actual total body weight. However, since this scaling may result in the administration of large doses of remimazolam, with risks of hemodynamic adverse consequences in the overweight patients, several weight scales must be developed to avoid excessive dosing in overweight patients. A current phase III trial for general anesthesia in Europe (NCT 03661489) explores the possibility of weight-independent dosing.

#### Elderly age and plasma albumin concentration

In this study, advanced age appeared to increase the time to extubation when remimazolam was administered on weight basis. One possibility is increased brain sensitivity to remimazolam as well as other benzodiazepines observed in the elderly [11–13], considering that no changes in PK with age were demonstrated in the use of remimazolam [9]. However, the possibility of higher plasma remimazolam concentration due to decreased cardiac output with less volume of distribution in the elderly cannot be ruled out. This results in slower hepatic circulation with subsequent reductions in drug metabolism and the clearance rate.

Midazolam, another benzodiazepine with short-term effects, also becomes increasingly potent with age and is influenced by sex. Sun et al. [14] reported that young females required a higher dose, and that with age, lower doses were indicated, which might be due to female hormones affecting the GABA receptors in the hippocampus and other areas of the limbic system. With regard to remimazolam, clearance was 11% higher in females than in males [9], consistent with a 3–5 min faster time to extubation in females [15], although the current study showed no sex-related differences.

Our analysis revealed as well that patients with lower plasma albumin concentration had longer times to extubation, suggesting another possibility on the effect of aging. Like other benzodiazepines, plasma protein binding of remimazolam was > 91% [16], primarily to human serum albumin. Because its unbound pharmacologically active fraction crosses the brain blood barrier, even a small decrease in plasma binding albumin might produce large changes in remimazolam’s effect. Furthermore, since we demonstrated an inverse relationship between age and plasma albumin concentration (r = -0.724), lower plasma albumin concentration might contribute to a longer time to extubation in the elderly. The decreases in volume of distribution, clearance rate, and plasma albumin binding result in a higher free plasma concentration of remimazolam. Thus, lower remimazolam doses may be warranted for elderly patients similarly to other anesthesia drugs.

### Relationship between predicted remimazolam concentration and time to extubation

Although the drug’s effect is dependent on its binding site concentration as a function of dose and time, responses to specific drug concentrations are difficult to predict especially in clinical use. Therefore, assuming that remimazolam is free and active, we generated a dose-response curve using a logarithmic X-axis scale (predicted remimazolam concentration) and a linear Y-axis scale (central nervous system effect). Because remimazolam is a full agonist of GABA_A_ receptors and elicits a maximal response, on a semi log scale it is easier to detect EC_50_ using sigmoidal dose-response curves.

As shown in a schematic representation of the logarithmic dose-response curve, the log predicted remimazolam concentration at the time of extubation is at a time when all the patients become awake enough to be extubated and have attained awareness and the same central nervous system effect. The LPF(-) group represented a significantly lower log predicted concentration than that exhibited by the SP group at the time of extubation. These data indicate that remimazolam in the LPF(-) group has a higher potency and/or efficacy than those of the SP group by shifting the dose-response curve to the left (Fig 3). Nevertheless, there was no difference in the log predicted concentrations at the end of infusion between the SP and LPF(-) groups. Thus, the LPF(-) group demonstrated a significantly greater difference between the log predicted remimazolam concentration at the end of infusion and that upon extubation than that demonstrated by the SP group. Because the difference in the log predicted concentration is proportional to that in the central nervous system effect, the change in remimazolam-produced response in the LPF(-) group is greater than that in the SP group as seen in the dose-response curve (Fig 3). These results suggest that amplified responses to remimazolam in the LPF(-) group (Fig 3) may result in excessively deep anesthesia at the end of its infusion when its dosage is solely based on total body weight in the LPF(-) group.

Amplification is an important factor of pharmacologic responses where only a minute quantity of the drug can modify biologic responsiveness at the sites in the drug-receptor coupling and following signal transduction system. Elderly people seem to be more sensitive to the effects of benzodiazepines on the central nervous system, mainly attributed to PK changes. However, since clinical studies have demonstrated that the impact of aging is much greater than could be expected from changes in PK alone, amplification of remimazolam response may also be ascribed to alterations in PD behaviors.

### Comparison with propofol

In addition to remimazolam, propofol is a short-term intravenous anesthetic with very rapid onset and offset. It is rapidly metabolized in the liver and at extrahepatic sites, has a shorter half-life, and does not accumulate. We have previously collected data on the time to extubation in 595 patients (females/males: 345/250; BMI: 23.2 kg/m^2^ [20.8–26.8 kg/m^2^]) who underwent surgery and received intravenous propofol-remifentanil total anesthesia. The time to extubation was 9.8 min (6.3–15 min), whereas in this study, where remimazolam was used, the time to extubation was 13.0 min (8–20 min) (all data represent median [25–75% interquartile range] values). Thus, a prolonged time to extubation, defined as 15 min or longer, seems appropriate for general anesthesia using either propofol or remimazolam. We have no data regarding which anesthetic offers faster wake-up time.

Kilpatrick et al. [17] reported that remimazolam differs from propofol in terms of its low risk of cardiovascular and respiratory depression and injection pain. Furthermore, the benzodiazepine antagonist flumazenil can reverse the effects of remimazolam in cases of adverse events and further reduce recovery time; no reversal agent is available for propofol. Propofol has several advantages over remimazolam as well. First, the availability of ready-to-use propofol formulations could enhance its use in general anesthesia. Remimazolam is a lyophilized powder requiring reconstitution, and the formula for reconstitution in the USA and EU can be considered excessively complicated; however, the formula for reconstitution available in Japan is quite simple. Second, over the past decade, increased attention has been paid to controlling the cost of anesthesia. Since institutions tend to limit the use of more expensive drugs, practitioners may be confronted by the cost not only of remimazolam, but also of flumazenil. For long-duration general anesthesia using remimazolam, the anesthetic drug cost in Japan may be about 1.5 times or more compared to that of propofol. Wanderer et al. [18] investigated the significant contributors to variation in anesthetic drug cost and concluded that the overall cost of anesthetic drugs was low and that cost-saving efforts may be better focused elsewhere.

### Limitations

There are limitations of the trial that need to be addressed. First, in the current study, all patients were Japanese. In Japan, the ideal body weight is designated as a BMI of 22 kg/m^2^ and obesity as BMI ≥ 25 kg/m^2^. The WHO classifies individuals with a BMI ≥ 25 kg/m^2^ as overweight, and those with BMI ≥30 kg/m^2^ as obese. Although the incidence of severe obesity is considerably rare in Japan compared to that in Western countries, the prevalence of overweight-related diseases is comparable. Moreover, although it appears to be reasonable that this study indicated BMI > 22 kg/m^2^ as a factor in increasing the time to extubation, further investigations in Western countries are needed for revising the dosing of remimazolam.

Second, co-administrated opioids might influence the time to extubation. In this study, remifentanil, an opioid with extraordinarily fast clearance, was administered to all patients, leading to a well-known synergistic potentiation of benzodiazepine-induced sedation and respiratory depression [19–21]. We considered the absence of opioid effects as the following conditions: 1) spontaneous breathing ≥ 8 breaths/min, 2) spontaneous breathing < 8 breaths/min, in principle, with predicted effect-site concentrations of remifentanil or fentanyl ≤ 2 ng/mL, and ≤ 1.5 ng/mL in the elderly.

Third, remimazolam has been used for general anesthesia in Japan, whereas the use of this drug is limited for procedural sedation less than 30 min in the USA and almost other countries. Our conclusions using long-duration of general anesthesia may not correspond exactly to data on short-duration of procedural sedation.

## Conclusions

In general anesthesia with remimazolam, the time to extubation was affected by BMI, age, and plasma albumin concentration. Lower remimazolam doses can be considered for some medically complex cases such as patients who are overweight patients, the elderly, or those with lower plasma albumin concentration.

## Supporting information

S1 File Dataset underlying the findings. (PDF)
