# Supplementary material for: Factors affecting prolonged time to extubation in patients given remimazolam
Source: PLoS One. 2022 May 18;17(5):e0268568. doi: 10.1371/journal.pone.0268568 (PMC9116664; doi:10.1371/journal.pone.0268568)
Supplement: S1 File — (PDF) [file pone.0268568.s001.pdf]

Patient No.	Sex	Age	Height	Body weight	BMI	Plasma albumin	ASA class
1	M	44	174.5	76.7	25.18863	4.8	1
2	M	77	163	49.6	18.66837	3	2
3	F	70	151.5	41.1	17.90674	4.3	3
4	F	55	158.8	72.8	28.86891	4.5	2
5	F	26	169.2	64.3	22.46003	4	2
6	M	61	174	93.6	30.91558	4.4	2
7	M	73	155	48.5	20.1873	3.5	2
8	F	72	152.5	54.9	23.60656	4.4	1
9	F	57	151.7	49.6	21.55314	4.4	2
10	F	48	145	40	19.02497	4.3	1
11	M	72	166	56.6	20.53999	3.6	3
12	M	82	153.1	47	20.05153	3	3
13	F	44	159.4	48	18.89142	4.1	2
14	F	69	148	56.4	25.74872	3.2	2
15	M	24	169	58	20.30741	4.4	1
16	M	79	165.3	62.6	22.91019	2.8	2
17	F	86	155.7	63.4	26.15243	2.5	2
18	F	68	151.5	45.6	19.86733	4	2
19	M	58	161.9	65	24.79817	4.3	2
20	F	75	144.2	45.5	21.88169	3.2	2
21	M	57	170.4	67.5	23.24688	4.6	2
22	M	78	165	61	22.40588	3.7	2
23	M	72	153.3	49.8	21.19069	4.6	2
24	M	87	158.7	67.3	26.72152	2.4	2
25	F	92	150	39.2	17.42222	2.4	3
26	M	58	170.4	61.4	21.14605	3.8	2
27	M	60	163.8	86.4	32.20223	4.1	2
28	M	80	157	83	33.67277	2.8	3
29	F	84	143.7	43.5	21.06569	3.5	3
30	M	33	173	54.4	18.17635	4.3	1
31	F	87	146.5	52.8	24.60133	3.5	3
32	F	67	155.8	59.3	24.42979	3.7	2
33	F	51	163.5	82.3	30.78678	4.2	2
34	M	73	167.5	64.1	22.84696	4.1	2
35	F	53	162	68	25.91068	3.6	3
36	M	81	157	56.7	23.00296	2.9	3

37	F	81	140	50	25.5102	2.2	3
38	M	43	176.4	90.3	29.01954	3.6	3
39	M	82	156.5	64.1	26.17154	3.6	2
40	F	88	150	57.9	25.73333	2.9	2
41	M	87	152	56.7	24.5412	3.3	3
42	M	59	177	80.8	25.7908	3.5	2
43	M	72	169.5	84.7	29.48112	3.2	2
44	F	55	159.5	107.7	42.33449	4.4	2
45	F	90	138	42.3	22.21172	2.8	3
46	F	61	155.6	60.7	25.07088	3.9	1
47	M	79	165.5	75.5	27.56455	2.8	2
48	F	76	153.2	72.4	30.84758	3.2	2
49	F	75	155	46.4	19.31322	2.7	3
50	M	70	174	94	31.04769	3.2	2
51	M	16	159	60	23.73324	4.5	2
52	F	84	157	54.3	22.02929	1.3	3
53	F	80	138.4	42.5	22.18793	2.3	3
54	F	86	139.1	61.8	31.93995	2.7	3
55	F	85	143.5	70.2	34.0905	2.4	2
56	M	65	165	104.4	38.34711	2.3	3
57	F	81	146	37	17.35785	2.8	2
58	M	80	162.2	80.6	30.63609	4.3	2
59	M	84	167.5	66.4	23.66674	3.3	3
60	F	63	160.5	60.5	23.4858	3.6	2
61	F	96	147.1	62.1	28.69897	3.5	2
62	F	52	157	42.7	17.32322	4.6	2
63	M	79	157.7	53.4	21.47227	4	3
64	M	88	161.3	50.1	19.25613	2.6	3
65	F	85	139.6	43.8	22.47518	2.5	2

Duration of surgery	Duration of anesthesia	Dose of remimazolam	Adiministration of flumzenil,1	Time to extubation	remimazolam concentration at the end of infusion	remimazolam concentration at the time of extubation	Difference of concentration
167	206	99.58	0	2	0.315003	0.202248	0.112755
264	302	70.98	0	3	0.259177	0.199029	0.060148
32	64	18.47	0	3	0.359482	0.222646	0.136836
252	337	110	0	4	0.172744	0.096526	0.076218
169	198	126.18	0	5	0.569181	0.35205	0.217131
219	281	147.1	0	5	0.278931	0.134465	0.144466
44	81	42.28	0	5	0.360073	0.264337	0.095736
58	94	32.22	0	6	0.220775	0.132276	0.088499
68	106	36.3	0	6	0.23387	0.145035	0.088834
339	380	147.53	0	6	0.470592	0.299996	0.170596
367	438	109.95	0	7	0.211498	0.130809	0.080688
142	203	239.62	0	7	0.451819	0.146347	0.305472
95	128	92.08	0	7	0.645578	0.345154	0.300423
111	139	49.67	0	7	0.240857	0.158714	0.082143
117	154	182.55	0	7	0.585728	0.41412	0.171608
103	136	40.4	0	8	0.181156	0.094622	0.086534
174	232	79.75	0	8	0.218705	0.104424	0.114281
97	177	42.08	0	8	0.149039	0.094372	0.054667
149	188	133.13	0	9	0.538274	0.277291	0.260983
163	213	92.87	0	9	0.35968	0.198689	0.160991
93	157	69.08	0	9	0.335849	0.160981	0.174868
162	247	59.14	0	10	0.153047	0.101228	0.051818
122	156	66.05	0	10	0.390651	0.210555	0.180096
250	293	98.1	0	10	0.255764	0.12284	0.132924
92	152	27.08	0	10	0.216762	0.125899	0.090862
96	138	52.8	0	11	0.354538	0.156869	0.197669
150	211	81.33	0	11	0.242352	0.108118	0.134233
149	200	88.98	0	11	0.215999	0.06924	0.146759
38	72	21.92	0	11	0.306883	0.127656	0.179227
349	397	249.42	0	12	0.819262	0.34883	0.470433
251	302	92.9	0	12	0.288535	0.130062	0.158473
59	121	51.75	0	13	0.2763	0.147726	0.128575
80	132	56.75	0	13	0.155713	0.085623	0.07009
199	232	55.64	0	14	0.245101	0.090769	0.154332
53	116	78	1	15	0.354973	0.170509	0.184464
200	246	63.2	1	15	0.207213	0.084772	0.12244

40	72	18.31	0	16	0.183381	0.08438	0.099001
260	348	156.17	1	16	0.301698	0.102735	0.198963
75	157	44.08	0	17	0.221001	0.07836	0.142642
59	118	44.61	0	17	0.216719	0.103586	0.113133
59	112	45.43	0	17	0.259144	0.139382	0.119762
233	331	185.67	1	18	0.347866	0.136174	0.211693
188	253	92.58	0	18	0.149295	0.070404	0.078891
31	61	30.42	0	19	0.142099	0.055361	0.086738
127	245	47.75	1	19	0.206303	0.098709	0.107594
295	348	191.8	0	19	0.303054	0.158979	0.144075
295	375	137.87	0	20	0.194099	0.067078	0.127021
343	416	172.05	0	20	0.200516	0.088886	0.11163
81	163	62.1	1	20	0.466247	0.162206	0.304042
151	207	233.62	1	20	0.276624	0.158353	0.11827
73	124	67.08	1	21	0.579725	0.174291	0.405434
130	215	116.57	1	21	0.373788	0.19228	0.181507
37	114	37.73	1	21	0.409399	0.157391	0.252008
37	132	46.81	1	22	0.353226	0.116431	0.236795
192	309	118.95	0	23	0.124302	0.056558	0.067744
314	409	189.33	0	23	0.176771	0.056296	0.120475
14	74	16.87	1	23	0.410582	0.103242	0.307339
118	169	110.08	0	24	0.290687	0.100482	0.190205
209	261	100.85	0	25	0.298709	0.100924	0.197785
45	86	40.4	0	26	0.482722	0.111248	0.371474
47	100	32.5	1	28	0.197931	0.070363	0.127568
92	152	60.3	1	31	0.389024	0.116642	0.272383
27	96	30.17	0	32	0.329661	0.083882	0.245779
320	390	109.33	1	36	0.235578	0.069311	0.166267
276	399	75.67	1	40	0.18952	0.051149	0.138371

logM at the end of infusion	logM at the time of extubation	Differenc of logM
-6.27769	-6.47012	0.192431
-6.3624	-6.47708	0.114681
-6.22032	-6.42838	0.208062
-6.5386	-6.79136	0.25276
-6.02075	-6.2294	0.208646
-6.3305	-6.64739	0.316888
-6.21961	-6.35384	0.134233
-6.43205	-6.65452	0.222469
-6.40703	-6.61453	0.207501
-6.10336	-6.29888	0.195529
-6.45069	-6.65936	0.208667
-6.12104	-6.61062	0.48958
-5.96605	-6.23799	0.271935
-6.39424	-6.57539	0.181145
-6.0083	-6.15887	0.15057
-6.51795	-6.80001	0.28206
-6.43614	-6.7572	0.321058
-6.6027	-6.80116	0.198458
-6.045	-6.33306	0.288067
-6.22008	-6.47783	0.257743
-6.24986	-6.56922	0.319369
-6.59118	-6.7707	0.179522
-6.18421	-6.45264	0.268424
-6.36816	-6.68666	0.318501
-6.44002	-6.67598	0.235959
-6.22634	-6.58046	0.354127
-6.39155	-6.7421	0.350547
-6.44155	-6.93564	0.494095
-6.28903	-6.66996	0.380932
-5.86258	-6.23339	0.37081
-6.3158	-6.66185	0.346047
-6.33462	-6.60654	0.271925
-6.58368	-6.84341	0.259733
-6.38666	-6.81806	0.431408
-6.2258	-6.54425	0.318448
-6.45958	-6.84775	0.388162

-6.51265	-6.84976	0.337114
-6.29643	-6.76428	0.467856
-6.43161	-6.88191	0.450302
-6.4401	-6.7607	0.320595
-6.36246	-6.63179	0.269334
-6.23459	-6.64191	0.40732
-6.60195	-6.9284	0.326448
-6.62341	-7.0328	0.40939
-6.46149	-6.78164	0.320147
-6.29448	-6.57466	0.28018
-6.48798	-6.94942	0.461443
-6.47385	-6.82717	0.353317
-6.10738	-6.56593	0.45855
-6.33411	-6.57637	0.242262
-6.01278	-6.53473	0.521947
-6.20338	-6.49207	0.28869
-6.16385	-6.57902	0.415167
-6.22795	-6.70993	0.481983
-6.68152	-7.02351	0.341985
-6.52859	-7.02552	0.496931
-6.1626	-6.76214	0.599541
-6.31257	-6.77391	0.461336
-6.30075	-6.77201	0.471255
-6.0923	-6.72971	0.637403
-6.47949	-6.92866	0.44917
-6.18602	-6.70915	0.523123
-6.25793	-6.85233	0.5944
-6.40386	-6.9352	0.531333
-6.49835	-7.06716	0.568816
